# A cross-sectional and longitudinal study of how two intervention methods affect the anxiety, sleep quality, and physical activity of junior high school students under quarantine

**DOI:** 10.3389/fpsyg.2023.1099732

**Published:** 2023-06-23

**Authors:** Peng Chen, Ying Chen, Shengjie Jin, Pengcheng Lu

**Affiliations:** ^1^College of Physical Education, Yancheng Teachers University, Yancheng, China; ^2^College of Foreign Languages, Wuhan University of Science and Technology, Wuhan, China; ^3^College of Physical Education, Yangzhou University, Yangzhou, China

**Keywords:** psychological nursing, physical activity, sleep quality, anxiety, junior high school student, cross-sectional-longitudinal study

## Abstract

**Purpose:**

This study investigated levels of anxiety and sleep quality and their association with physical activity in junior high school students under quarantine during the COVID-19 pandemic. It also tests the effectiveness of physical activity and psychological nursing interventions in alleviating anxiety ‘and improving sleep quality.

**Methods:**

In July 2021, 14,000 home-quarantined junior high school students in Yangzhou City (China) were selected by random cluster sampling to complete an online survey. We then selected 95 junior high school students for an 8-week longitudinal experiment exploring whether the two types of intervention made positive contributions to students' anxiety, sleep quality, and physical activity.

**Results:**

The cross-sectional study revealed that physical activity was significantly related to anxiety and sleep quality. In the longitudinal study, students who underwent the exercise intervention or the psychological nursing intervention experienced significant improvement in their anxiety levels. The exercise intervention also promoted improved sleep quality. Overall, the exercise intervention was more effective than the psychological nursing intervention in reducing levels of anxiety and sleep disorders.

**Conclusion:**

During the epidemic period, junior high school students should be encouraged to spend more time engaging in physical activity, and their sleep quality and anxiety shouldbe focused on.

## 1. Introduction

Against the background of the normalization of the COVID-19 epidemic in China, the rapid spread of virus mutations necessitates a range of prevention and control measures, including closing workplaces and schools. Residents in affected areas have been required to follow quarantine measures, resulting in negative impacts on their daily life and physical and mental health. One group particularly vulnerable to the detrimental effects of home or hotel quarantine, especially with respect to psychology, sleep, and learning, is junior high school students (Tao et al., 2023). With rising incidences of anxiety and depression in children and adolescents subject to sudden quarantines, there is an urgent need for targeted interventions to improve their physical and mental health (Önder et al., 2022). Therefore, to promote the healthy development of the adolescent population, it is especially important to focus on the physical and mental health problems they experience during COVID-19 quarantine periods, including what factors can worsen or alleviate these problems (Houghton et al., 2022).

Traditional approaches to the prevention and early treatment of common mental health diseases include physical therapy, psychotherapy, and drug therapy, but their effectiveness is somewhat limited by factors such as poor compliance and the side effects of drugs (Samushiya et al., 2021). Exercise intervention is increasingly recognized as an effective treatment for depression because it is easy to regulate and has no obvious side effects. By adjusting the timing, type, and organizational form of exercise intervention, the interests and hobbies of different depressed people as well as the time and frequency of exercise intervention are divided into different exercise items for intervention (Schuch and Stubbs, 2019). The influence of exercise interventions on sleep quality may be related to circadian rhythms. The circadian rhythm system of the human body is mainly concentrated in the suprachiasmatic nucleus of the hypothalamus and adjacent sites. Circadian signals are transmitted to multiple sleep-and-wake brain regions via the suprachiasmatic nucleus to regulate the phase transitions of each sleep stage and the transition between sleep and wake cycles. Disturbance of circadian rhythms and amplitude variation are the main causes of abnormal sleep structure. With the development of modern medicine, increasing attention is being paid to the mental health and happiness of different populations. Research has found that psychological nursing can alleviate low mood in students. Moreover, positive psychological interventions appear to have a calming and comforting effect on patients with schizophrenia. However, there are few reports of such interventions being targeted at primary and middle school students (Singh et al., 2020). Although academic circles have different perspectives on the hot topic of various sports and sports combined intervention in psychological problems, traditional sports may no longer be the most effective exercise for alleviating depression in students; instead, different forms of intervention activities may be more therapeutic.

To address gaps in the literature, this study investigates the influence of two types of intervention—exercise and psychological nursing—on the anxiety and sleep quality of junior high school students under home quarantine. We hope that our findings will offer insights on effective social support for adolescents during the COVID-19 epidemic, as well as providing a theoretical basis for improving adolescent anxiety and sleep quality.

## 2. Subjects and methods

### 2.1. Subjects of the survey

For the cross-sectional survey conducted between July and September 2021, we selected 14,000 junior high school students in Yangzhou City, Jiangsu Province, using stratified cluster random sampling. All participants were aged 12–15 years old and were in the first to the third grade. We sought to ensure roughly equal representation of male and female students and urban and rural residents. All participants gave fully informed to participating before completing the questionnaire. A total of 12,927 valid questionnaires were returned, representing an effective response rate of 92.3%. The data were analyzed using chi-square tests and logistic regressions. Table 1 presents basic information on the final sample.

**Table 1:** Basic information of participants.

**Category**	**Items**	***n***	**%**
Gender	Male	6,230	48.19
	Female	6,698	51.81
Grade	First	4,364	33.76
	Second	4,268	33.01
	Third	4,296	33.23
BMI	Underweight	2,117	16.38
	Normal	8,234	63.69
	Overweight	1,902	14.71
	Obese	675	5.22
Only child?	Yes	4,333	33.52
	No	8,595	66.48
Urban or rural?	Urban	7,859	60.79
	Rural	5,069	39.21
Living mode	With parent(s)	9,023	69.79
	With relative(s)	3,167	24.50
	Trusteeship	738	5.71
Family structure	Two-parent	11,768	91.03
	One-parent	769	5.95
	Recombination	391	3.02
Total		12,927	100

For the longitudinal experiment, which started in September 2021, we selected 95 home-quarantined junior high school students aged 12–15 years old as participants: 75 had anxiety standard scores >4, while the other 20 had scores of 4 or below. Seventy-five participants with elevated anxiety were randomly divided into the anxiety control group (25 people), psychological nursing group (25 people), and exercise group (25 people). The experimental process was divided into pre-test and post-test. All participants took part voluntarily and were free to withdraw at any time. Before the start of the study, we obtained informed consent from the school teachers, participating junior high school students, and their parents. The study design was approved by the Ethics Committee of the Nursing School of Yangzhou University (Batch number: YXYLL-2020-106).

### 2.2. Measurements

#### 2.2.1. General information questionnaire

The general information questionnaire covered participants' gender, age, grade, body mass index (BMI), only-child status, urban–rural residence, living mode, and family structure. BMI was calculated by dividing weight by height^2^ (kg/m^2^). Overweightness and obesity were defined according to gender and age BMI thresholds in the national health standard Screening for Overweight and Obesity of School-age Children and Adolescents (WS/T 568-2018) (Chen et al., 2018). For BMI, < 18.5 kg/m^2^ means underweight, 18.5–23.9 kg/m^2^ is normal, 24.0–27.9 kg/m^2^ signifies overweight, and ≥28.0 kg/m^2^ denotes obesity.

#### 2.2.2. Generalized Anxiety Scale

We used Generalized Anxiety Scale (GAD-7) to evaluate participants' anxiety symptoms. The scale comprises seven items, each scored from 0 to 3. Total scores of 0–4 indicate minimal anxiety, 5–9 points represent mild anxiety, 10–14 denote moderate anxiety, and 15–21 suggest severe anxiety. Cronbach's α coefficient of the GAD-7 in this study was 0.932.

#### 2.2.3. Physical Activity Rating Scale

The revised Physical Activity Rating Scale (PARS-3) by Liang Deqing was employed to assess the amount of physical activity of junior high school students (Zeng et al., 2022). The scale is divided into three items: exercise intensity, time, and frequency. Each item is answered on a five-point scale from 1 to 5, and exercise amount = intensity × (time−1) × frequency. Following the grade classification criteria used by Xia Xiangwei, a total score of ≤ 4 points denotes no exercise, 5–19 points indicate a small amount of exercise, 20–42 points represent a medium amount, and 43 or more points suggest a large amount. The scale has been shown to have high reliability and validity, with a test–retest reliability of 0.82. In the present study, Cronbach's α value of the scale was 0.639.

#### 2.2.4. Pittsburgh Sleep Quality Index Questionnaire

We used the Chinese version of the Pittsburgh Sleep Quality Index Questionnaire (PSQI), as translated by Zou et al. (2023). The scale comprises seven items, each scored from 0 to 3 points, meaning the total score ranges from 0 to 21. Regarding the total PSQI score, ≤ 5 indicates good sleep quality, 6–10 means average sleep quality, 11–15 represents poor sleep quality, and 16–21 suggests very poor sleep quality. In international tests, the PSQI has demonstrated good reliability and validity. Cronbach's α coefficient of the scale in this study was 0.84.

### 2.3. Experimental process

The sample size for the longitudinal experiment was determined using Bayesian factor analysis, and the Bayesian factor threshold BF10 for stopping data collection was set at 10 (threshold for accepting H_1_) and 1/10 (threshold for accepting H_0_). Specifically, to test whether moral valence impacts on matching attempts in perceptual matching, this study focused on the difference in response time under different conditions and adopted Bayesian factor sequence design to test the BF values corresponding to three key effects: (1) the interaction between moral titer and matching degree; (2) the reaction time of the moral positive matching condition is faster than that of the neutral matching condition; and (3) the reaction time of the moral negative matching condition is slower than that of the neutral matching condition. The three key effects will be analyzed using Bayesian repeated-measures ANOVA and Bayesian paired-samples *t*-test (unilateral), respectively, which both use JASP built-in priors. Considering the practical factors of this study, the minimum sample size was set at 18 and the maximum sample size at 25 participants.

In the exercise group, the intervention lasted for 8 weeks and combined high-intensity interval training (HIIT) with regular exercise. The HIIT activities comprised 10 movements: 10 burpees, 30 opening and closing jumps, 10 kneeling push-ups, 20 alternating knee lifts, 20 squats, 20 bench bends, 10 squat jumps, 20 supine tummy rolls, 10 lunge squats, and 20 supine hip straightens on both sides. These exercises were performed at a medium intensity for 20 min, and completed 3 times per week. To ensure that exercise intensity was at the desired (safe) level, we randomly selected six participants to wear Polar heart rate monitors; their heart rates were recorded at quiet time and 3, 6, and 15 min into the exercise. Before the experiment, the research purpose and the exercise regime were clearly explained to all participants. They were forbidden to drink alcohol, coffee, tea, or any other drinks affecting sleep for 48 h before the experiment and instructed not to engage in strenuous exercise for 24 h before the experiment. No participants had any major life events.

In the psychological nursing group, quarantined students with elevated anxiety received remote online support from the nursing staff of Yangzhong People's Hospital. During the 8-week intervention, each student met remotely with a nurse once per week for 30–40 min. The intervention was designed to create a quiet and comfortable environment for dialogue, conducive to good communication; nurses maintained a cordial and warm attitude toward students and used technical language where appropriate, aiming to help students express their innermost thoughts and reduce their resistance to psychological intervention. Nurses explained to students the adverse effects of anxiety and how lack of physical activity is detrimental to physical and mental health, as well as providing information on the main causes of anxiety among junior high school students, such as learning pressure, change of environment, and separation. They also paid close attention to emotional variations in students, and gave targeted guidance based on each student's anxiety level, seeking to reduce their psychological burden and affirm their efforts in the intervention process. In addition, the nurses gave timely examples to illustrate the radical and fluctuating nature of anxiety, thereby aiming to lessen students' fear and concerns and promote their confidence. Students also received training in relaxation methods, such as listening to soft music to relax their minds and reduce both psychological tension and anxiety. Finally, the nurses also encouraged students to actively exercise at home.

Participants in the anxiety control group and the normal control group lived a normal home life and engaged in study as usual.

### 2.4. Statistical analysis

The Kolmogorov–Smirnov normality test was conducted on the questionnaire results. *p*-values for anxiety, sleep quality, and total questionnaire all exceeded 0.05. At the test level of α = 0.05, we can conclude that the survey results of each dimension and the total questionnaire did not significantly differ, indicating bivariate normal distributions suitable for Pearson correlation analysis. As *p*-value of the attitude dimension was < 0.001, Spearman correlation analysis was used.

The data were analyzed using SPSS 26.0 software. For MPAI, total scores of 34–50 represent mild cell phone addiction, 51–68 denote moderate addiction, and 69–85 indicate severe addiction. Paired-samples *t*-tests were used to compare the data before and after the intervention, and independent-samples *t*-tests and one-way ANOVAs were used to test the different groups.

## 3. Results

### 3.1. Comparative levels of anxiety and sleep quality according to demographic characteristics

Chi-square tests were performed to identify any significant differences in the levels of anxiety and sleep quality within demographic categories. The results indicate that anxiety (*p* < 0.05) and sleep quality (*p* < 0.01) significantly differed among participants of different grades during the quarantine period. Anxiety also significantly differed between urban and rural dwellers, while sleep quality significantly differed between male and female students. No other significant differences were found. The full results are shown in Table 2.

**Table 2:** Chi-square test results for comparative levels of anxiety and sleep quality in demographic categories of junior high school students.

**Category**	**Items**	**Anxiety** ***n*** **(%)**		**Sleep quality** ***n*** **(%)**	
		**Without anxiety**	**With anxiety**	**Good**	**Bad**
Gender	Male	3,638 (28.12)	2,592 (20.05)	2,852 (49.77)	3,378 (46.93)
	Female	3,740 (28.93)	2,957 (22.87)	2,878 (50.23)	3,820 (53.07)
		χ^2^: 2.218	*p*: 0.136	χ^2^: 10.331	*p*: 0.001^**^
Grade	First	2,426 (32.89)	1,689 (30.41)	2,014 (35.15)	2,350 (32.65)
	Second	2,431 (32.95)	1,884 (33.96)	1,807 (31.54)	2,461 (34.19)
	Third	2,521 (34.16)	2,016 (35.63)	1,909 (33.32)	2,387 (33.16)
		χ^2^: 8.230	*p*: 0.016^*^	χ^2^: 12.740	*p*: 0.002^**^
Only child?	Yes	4,932 (66.84)	1,863 (33.56)	1,879 (32.79)	2,454 (34.09)
	No	2,446 (33.16)	3,686 (66.44)	3,851 (67.21)	4,744 (65.91)
		χ^2^: 0.087	*p*: 0.768	χ^2^: 2.421	*p*: 0.120
Urban or rural?	Urban	2,517 (34.11)	2,210 (39.82)	2,225 (38.83)	2,844 (39.51)
	Rural	4,861 (65.89)	3,339 (60.18)	3,505 (61.17)	4,354 (60.49)
		χ^2^: 16.864	*p*: 0.000^**^	χ^2^: 0.619	*p*: 0.431
Living mode	With parent(s)	5,198 (70.46)	3,869 (69.72)	3,956 (69.04)	5,067 (70.39)
	With relative(s)	1,734 (23.51)	1,366 (24.61)	1,445 (25.22)	1,722 (23.92)
	Trusteeship	446 (6.02)	314 (5.67)	329 (5.74)	409 (5.68)
		χ^2^: 0.968	*p*: 0.616	χ^2^: 3.042	*p*: 0.219
Family structure	Two-parent	6,773 (91.80)	5,045 (90.93)	5,235 (91.36)	6,533 (90.76)
	One-parent	407 (5.52)	332 (6.00)	330 (5.76)	439 (6.10)
	Recombination	198 (2.69)	172 (3.07)	165 (2.88)	226 (3.14)
		χ^2^: 1.177	*p*: 0.555	χ^2^: 1.459	*p*: 0.482

^*^p < 0.05.

^**^p < 0.01.

### 3.2. Correlations and regressions of physical activity with anxiety and sleep quality

As shown in Table 3, Pearson correlation analysis revealed that physical activity was correlated with anxiety (*r* = −0.174, *p* < 0.001) and sleep quality (*r* = −0.258, *p* < 0.001). Next, we used a simple linear regression model to analyze the impact of physical activity (independent variable) on anxiety and sleep quality (dependent variables). The model formulas were as follows:


$$\begin{array}{lll} \text{Anxiety } & = & 10.954\;-\;0.054{\;}^{*}\text{ physical activity }\\ \text{Sleep quality } & = & 9.074\;-\;0.087{\;}^{*}\text{ physical activity } \end{array}$$

**Table 3:** Pearson correlations of physical activity with anxiety and sleep quality.

		**Physical activity**
Anxiety	Correlation coefficient	−0.174^**^
	*p*	0.000
Sleep quality	Correlation coefficient	−0.258^**^
	*p*	0.000

^**^p < 0.01.

with respective *R*^2^ values of 0.030 and 0.066, indicating that physical activity explains the reasons of 3.0 and 6.6% changes in anxiety and sleep quality. Both regressions have overall significance (anxiety: *F* = 402.645, *p* = 0.000 < 0.05; sleep quality: 919.384, *p* = 0.000 < 0.05). The regression results are presented in Table 4.

**Table 4:** Linear regressions of sleep quality and anxiety on physical activity.

	**Unstandardized coefficients**		**Standardized coefficients**	***t***	***p*-value**	**VIF**	***R* ^2^**	**Adjusted *R*^2^**	***F***
	***B***	**Standard error**	β						
Constant	9.074	0.091	–	99.533	0.000^**^	–	0.066	0.066	*F*_(1, 12, 926)_ = 919.384, *p* = 0.000
Sleep quality	−0.087	0.003	−0.258	−30.321	0.000^**^	1.000			
Constant	10.954	0.086	–	127.395	0.000^**^	–	0.030	0.030	*F* (1,12,926) = 402.645, *p* = 0.000
Anxiety	−0.054	0.003	−0.174	−20.066	0.000^**^	1.000			

D-W value of sleep quality: D-W value of anxiety.

^**^p < 0.01.

### 3.3. Comparative levels of anxiety, physical activity, and sleep quality among different groups of junior high school students

As shown in Table 5, the results of independent-samples *t*-tests revealed significant differences in anxiety, physical activity, and sleep quality between the normal control group and the anxiety control group. The results illustrate that levels of anxiety, physical activity, and sleep quality significantly improved after the exercise intervention and psychological nursing during the quarantine period.

**Table 5:** Results of independent-samples *t*-tests comparing levels of anxiety, physical activity, and sleep quality between the normal control group and the anxiety control group.

**Group (mean ±standard deviation)**	**Anxiety**	**Physical activity**	**Sleep quality**
Normal control group (*n* = 20)	4.84 ± 1.64	19.65 ± 7.67	7.75 ± 3.02
Anxiety control group (*n* = 25)	10.40 ± 3.73	21.87 ± 9.01	9.80 ± 3.78
*t*	−10.125	−1.390	−3.150
*p*	0.000^**^	0.007^**^	0.002^**^

^*^p < 0.05.

^**^p < 0.01.

A one-way ANOVA was performed to reveal differences between the anxiety control group, exercise group, and nursing group in levels of anxiety, physical activity, and sleep quality. As reported in Table 6, we found significant differences in anxiety, physical activity, and sleep quality between the three groups.

**Table 6:** ANOVA results for levels of anxiety, physical activity, and sleep quality in the three groups of students with elevated anxiety.

	**Group (mean** ±**standard deviation)**			***F***	***p*-value**
	**Anxiety control (*****n*** = **25)**	**Nursing (*****n*** = **25)**	**Exercise (*****n*** = **25)**		
Anxiety	6.84 ± 3.89	9.20 ± 2.64	14.42 ± 4.98	53.008	0.000^**^
Physical activity	27.29 ± 11.59	26.49 ± 9.30	31.13 ± 9.60	3.240	0.042^*^
Sleep quality	6.22 ± 3.81	9.56 ± 3.32	9.65 ± 2.85	18.822	0.000^**^

^*^p < 0.05.

^**^p < 0.01.

### 3.4. Pre- to post-intervention changes in levels of anxiety, physical activity, and sleep quality in each group

We carried out paired-samples *t*-tests to compare levels of anxiety, physical activity, and sleep quality in each group before and after the intervention. After the exercise intervention, there were significant improvements in student's levels of anxiety (*p* = 0.000), physical activity (*p* = 0.000), and sleep quality (*p* = 0.019). After the psychological nursing intervention, there was no significant difference in anxiety (*p* > 0.05), but there were significant differences in physical activity and sleep quality (*p* < 0.01). In both the anxiety control group and the normal control group, anxiety, sleep quality, and physical activity all significantly declined over the 8-week period. The full results are shown in Table 7.

**Table 7:** Comparative levels of anxiety, physical activity, and sleep quality within each group before and after intervention.

**Group**	**Measurement time**	***n***	**Statistical value**	**Anxiety**	**Physical activity**	**Sleep quality**
Exercise	Pre-intervention	25		10.58 ± 3.59	27.09 ± 9.38	10.75 ± 3.74
	Post-intervention	25		8.47 ± 2.91	31.13 ± 9.60	9.65 ± 2.85
			*t*	8.443	−5.338	2.414
			*p*	0.000^**^	0.000^**^	0.019^*^
Nursing	Pre-intervention	25		9.25 ± 2.82	30.15 ± 8.51	8.51 ± 3.78
	Post-intervention	25		9.01 ± 2.64	27.96 ± 7.59	9.56 ± 3.32
			*t*	0.256	2.407	−3.219
			*p*	0.014^*^	0.020^*^	0.002^**^
Anxiety control	Pre-intervention			6.84 ± 3.89	27.29 ± 11.59	6.22 ± 3.81
	Post-intervention			10.40 ± 3.73	21.87 ± 9.01	9.80 ± 3.78
			*t*	−12.445	6.293	−8.253
			*p*	0.000^**^	0.000^**^	0.000^**^
Normal control	Pre-intervention			2.27 ± 1.51	27.29 ± 11.59	5.44 ± 2.51
	Post-intervention			4.84 ± 1.64	19.65 ± 7.67	7.75 ± 3.02
			*t*	−10.427	6.747	−7.900
			*p*	0.000^**^	0.000^**^	0.000^**^

^*^p < 0.05.

^**^p < 0.01.

## 4. Discussion

The results demonstrated that, during the quarantine period, the incidence of anxiety among junior high school students was 43.0%, while 55.7% had sleep quality disorders. These levels are consistent with those reported by and Xu et al. (2019) and Chen and Qu (2021) for samples of junior high school students during the epidemic, but higher than those reported by Yao and Cao (2021) and Chen et al. (2022) in a normal environment. The epidemic is thus likely to explain differences in reported levels of anxiety and sleep quality. In our cross-sectional survey, anxiety was slightly more prevalent in female students (28.93%) than in male students (28.12%); bad sleep quality was also more prevalent in female students (53.07%) than in male students (46.93%). Anxiety prevalence was highest among third-grade students (35.63%), followed in turn by second-grade (33.96%) and first-grade students (30.41%); bad sleep quality was most prevalent in second-grade students (34.19%). Having one or more siblings, residing in a rural area, living with parents, and being in a two-parent family structure possessed significant anxiety and sleep quality problems.

Moderate exercise plays an important role in the physical and mental health development of adolescents (Biddle and Asare, 2011). According to the Guidelines for Physical Activity of Children and Adolescents in China, regular exercise can improve cardiovascular function, enhance metabolism, and improve muscle and bone health (Chen et al., 2020). The results show that the average level of moderate-to-vigorous physical activity among China's adolescents is only 37.66 min per day, far lower than the 60+ min per day recommended by the World Health Organization. In our study, the average physical activity level of junior high school students was only at a medium level; just 17.8% reported engaging in a high level of physical activity. Moreover, as BMI increases, the level of physical activity decreases, indicating that students who do not exercise are more likely to develop obesity. Male students reported significantly higher levels of physical activity compared with female students, consistent with the results of multiple prior studies. While in junior high school, children undergo rapid changes in physical form, physical function, and psychological state. In particular, boys experience rapid development in speed, strength, and endurance, resulting in greater gender differences in these physical attributes. Whereas male students often participate in sports and physical exercise in daily life, female students, due to physiological reasons, derive insufficient sense of self-efficacy from such activities, resulting in low enthusiasm for sports.

The level of self-reported physical activity was lowest in third-grade students, significantly lower than the levels reported by second- and first-grade students. In the third grade, students face heavy workloads and pressure to prepare for the high school entrance examination; devoting most of their time to study, they have little time available for physical exercise. At the end of the term, physical education is often given to the main class, resulting in insufficient physical education class hours, reduced activity time, and insufficient activity levels. We found that physical activity was negatively related to anxiety and sleep quality in junior high school students, indicating that the decline in physical activity under quarantine negatively impacted on their psychological wellbeing and sleep, due to the factors such as environment, online classes, and electronic devices. Therefore, our findings suggest that increasing physical activity levels could alleviate anxiety and improve the sleep quality of junior high school students.

Overall, the cross-sectional survey results indicate that junior high school students experienced an increase in anxiety, a drop in physical activity, and a decline in sleep quality while quarantined. One explanation for our findings is that junior high school students are a vulnerable group in public health emergencies: losing access to timely psychological guidance, they may be prone to developing negative psychological problems such as depression and anxiety. Another explanation is that these students usually engage in physical exercise on weekends after class or at school. Although it is possible to exercise at home, the influence of learning pressure, emergency issues, and students' consciousness may have contributed to a decline in their physical activity levels. Prior research has revealed that excessive use of electronic devices by junior high school students became a serious issue during the epidemic period; spending so much time looking at screens and remaining sedentary have been associated with anxiety, sleep disorders, and other physical and mental health problems. The anxiety of junior high school students during the epidemic urgently requires high vigilance and attention from society, schools, and responsible authorities (Bateman et al., 2016; Woods and Scott, 2016).

Adolescent mental health has always been a hot topic in China and abroad, with great importance to the nation, society, and families. Studies show that more than 30% of adolescents worldwide have mental health problems (Auerbach et al., 2018). Moreover, depression and anxiety are reportedly the third most common psychological problems in adolescents. Consistent with this study's results, depression levels rise with a decrease in physical activity: possible mechanisms include leptin resistance caused by low physical activity, anxiety induced by high levels of pro-inflammatory factors crossing the blood–brain barrier, and disturbance of intestinal flora. In our longitudinal experiment, junior high school students who underwent 8 weeks of exercise intervention or psychological nursing experienced a significant reduction in anxiety. The results thus demonstrate that both forms of intervention helped to alleviate students' anxiety, and the interventions were feasible and effective. The exercise intervention was also found to promote students' levels of physical activity and improve their sleep quality. By contrast, the psychological nursing intervention did not lead to improvements in levels of physical activity or sleep quality, which might be explained by the sedentarism and use of electronic devices that characterize learning through online courses. Therefore, students under quarantine should be encouraged to actively engage in physical exercise during the quarantine period. Moreover, schools and society should provide psychological guidance such as counseling, as well as conducting publicity campaigns and holding lectures on physical activity, sedentary behavior, and other related topics. These steps would help to reduce the pressure and difficulties of learning for junior high school students during the epidemic. In the home, parents should supervise how much time their children spend playing video games and pay attention to any changes in their children's psychological state. It is important that parents establish their own role models and drive students to engage more in physical exercise (Paolucci et al., 2018).

## 5. Limitations and future prospects

In recent years, depression has become considerably more common in student populations. With modern developments and lifestyle changes, traditional ways of treating depression have gradually lost their effectiveness, and exercise therapy has begun to be accepted by young patients. This study focused on junior high school students under quarantine status during the COVID-19 epidemic, resulting in several limitations. First, the online design of the experimental research made it difficult to control for various environmental factors likely to influence the measured variables. Moreover, we did not control for family structure and parenting behavior, which likely had a greater impact on students during the quarantine period compared with normal times. Second, the ratio of experimental staff to study participants was relatively large; in particular, the one-to-many approach of the psychological nursing intervention may have affected the experimental results, which consequently could not be well-promoted and applied. Third, longer term research is needed to investigate the volatility of anxiety in the target population. In addition, the causal associations between factors such as family support, diet, and anxiety levels require further study. Our findings suggest that exercise and psychological nursing interventions could be combined as a low-cost approach for reducing anxiety in junior high school students: future studies should test the effectiveness of this combination. Researchers should also test other innovative interventions for students and social families during the normalization of the epidemic.

## Data availability statement

The raw data supporting the conclusions of this article will be made available by the authors, without undue reservation.

## Ethics statement

The studies involving human participants were reviewed and approved by Ethics Committee of Nursing School of Yangzhou University (Batch number: YXYLL-2020-106). Written informed consent to participate in this study was provided by the participants' legal guardian/next of kin.

## Author contributions

PC and YC contributed to study conception and design, and to manuscript polishing, and revision. SJ and PL organized the database, performed statistical analysis, and wrote the first draft of the manuscript. All authors contributed to the article and approved the submitted version.
